# Experimental investigation of optically controlled topological transition in bismuth-mica structure

**DOI:** 10.1038/s41598-021-93132-9

**Published:** 2021-07-01

**Authors:** Anton Zaitsev, Dmitry Zykov, Petr Demchenko, Mikhail Novoselov, Ravshanjon Nazarov, Maxim Masyukov, Elena Makarova, Anastasiia Tukmakova, Aleksei Asach, Anna Novotelnova, Natallya Kablukova, Mikhail Khodzitsky

**Affiliations:** 1 Terahertz Biomedicine Laboratory, ITMO University, St. Petersburg, 197101 Russia; 2 Faculty of Energy and Ecotechnology, ITMO University, St. Petersburg, 197101 Russia

**Keywords:** Nanophotonics and plasmonics, Sub-wavelength optics, Two-dimensional materials

## Abstract

The hyperbolic materials are strongly anisotropic media with a permittivity/permeability tensor having diagonal components of different sign. They combine the properties of dielectric and metal-like media and are described with hyperbolic isofrequency surfaces in wave-vector space. Such media may support unusual effects like negative refraction, near-field radiation enhancement and nanoscale light confinement. They were demonstrated mainly for microwave and infrared frequency ranges on the basis of metamaterials and natural anisotropic materials correspondingly. For the terahertz region, the tunable hyperbolic media were demonstrated only theoretically. This paper is dedicated to the first experimental demonstration of an optically tunable terahertz hyperbolic medium in 0.2–1.0 THz frequency range. The negative phase shift of a THz wave transmitted through the structure consisting of 40 nm (in relation to THz wave transmitted through substrate) to 120 nm bismuth film (in relation to both THz waves transmitted through substrate and air) on 21 µm mica substrate is shown. The optical switching of topological transition between elliptic and hyperbolic isofrequency contours is demonstrated for the effective structure consisting of 40 nm Bi on mica. For the case of 120 nm Bi on mica, the effective permittivity is only hyperbolic in the studied range. It is shown that the in-plane component of the effective permittivity tensor may be positive or negative depending on the frequency of THz radiation and continuous-wave optical pumping power (with a wavelength of 980 nm), while the orthogonal one is always positive. The proposed optically tunable structure may be useful for application in various fields of the modern terahertz photonics.

## Introduction

The sign of real part of any material’s dielectric permittivity describes the nature of its electromagnetic response. The positive sign corresponds to dielectric materials which support propagating waves, while the negative one is responsible for metal-like media which reflect the radiation and support only evanescent penetrating field. At the same time, loss in both types of materials is determined by the imaginary part of a dielectric permittivity. However, there is another type of materials that combines both the mentioned ones: the tensor of their anisotropic dielectric permittivity consists of one negative and two positive diagonal components (I type of hyperbolic dispersion, rod-like structures, $\varepsilon _{xx}>0,\varepsilon _{yy}>0$, $\varepsilon _{zz}<0$) or two negative and one positive diagonal components (II type, layered structures, $\varepsilon _{xx}<0,\varepsilon _{yy}<0$, $\varepsilon _{zz}>0$)^1–4^:

1 $$\begin{aligned} \hat{\varepsilon }=\begin{pmatrix} \hat{\varepsilon }_{xx} &{} 0 &{} 0\\ 0 &{} \hat{\varepsilon }_{yy} &{} 0\\ 0 &{} 0 &{} \hat{\varepsilon }_{zz} \end{pmatrix}. \end{aligned}$$

**Figure 1:**
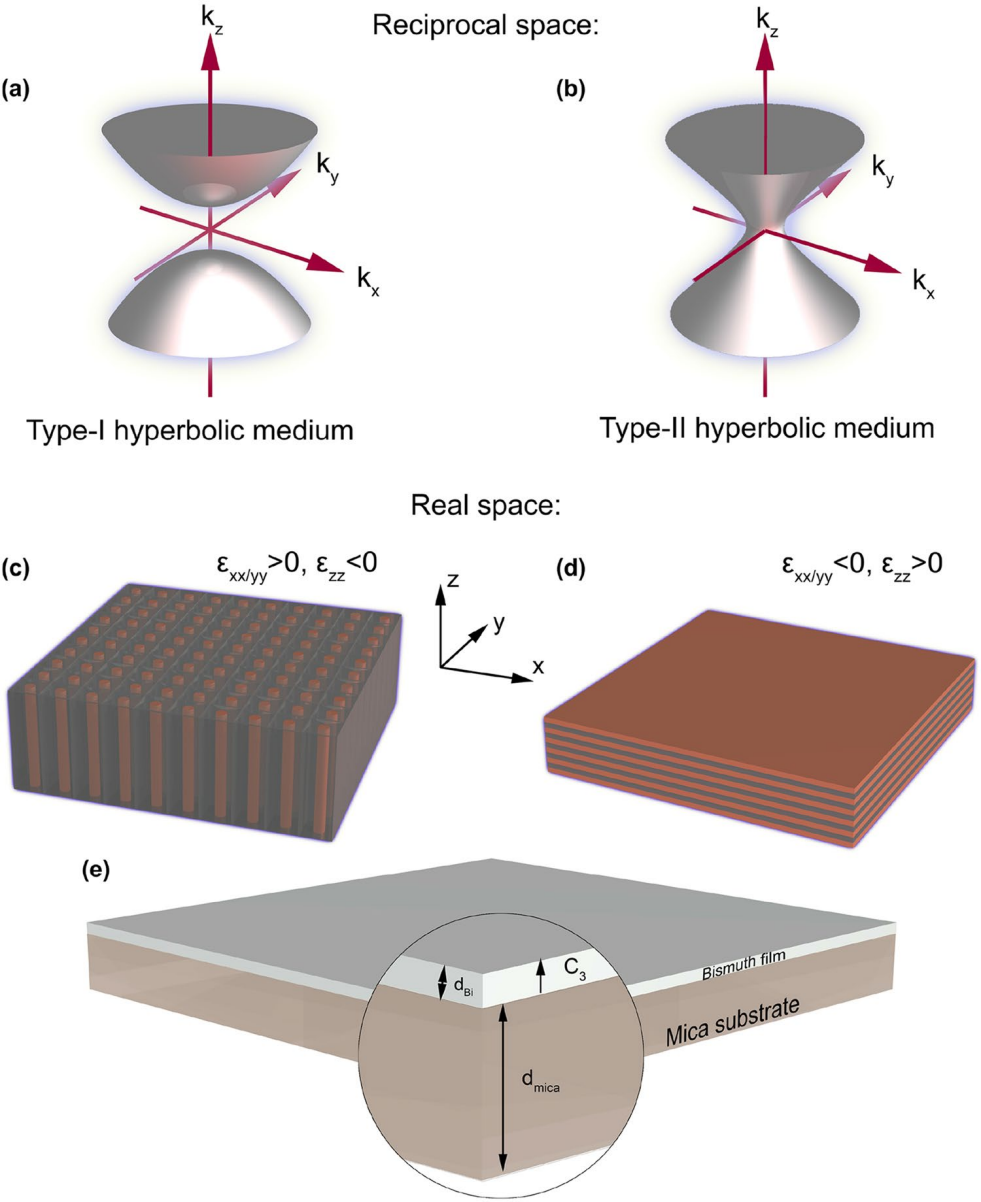
Isofrequency surfaces in reciprocal space (k-space) for I (**a**) and II (**b**) types of hyperbolic media and corresponding typical structural geometries (**c,d**). (**e**) The scheme of a layered structure proposed in this work (not in scale). The C$_{3}$ axis of a bismuth film is oriented normally to its plane.

Such media are based mainly on metal-like materials in which the motion of charge carriers is limited along some spatial directions. Thus, the behavior of an electromagnetic wave in hyperbolic media is more complex than in conventional media and is mathematically described by a dispersion relation:

2 $$\begin{aligned} \frac{k_x^2+k_y^2}{\varepsilon _{zz}}+\frac{k_z^2}{\varepsilon _{xx}}=\frac{\omega ^2}{c^2} , \end{aligned}$$

giving hyperbolic isofrequency surfaces in wave-vector space when $\varepsilon _{xx}=\varepsilon _{yy}<0$ or $\varepsilon _{zz}<0$ (Fig. 1a,b) for an electric uniaxial medium (the diagonal components of a permeability tensor are considered to be $\mu _{xx}=\mu _{yy}=\mu _{zz}=1$). In contrast, conventional materials are described with spherical or ellipsoidal isofrequency surfaces (dielectric-like media, $\varepsilon _{xx}>0$, $\varepsilon _{yy}>0$, $\varepsilon _{zz}>0$) or with empty k-space (metal-like media, $\varepsilon _{xx}<0$, $\varepsilon _{yy}<0$, $\varepsilon _{zz}<0$). The scheme of a studied structure is depicted in Fig. 1e, and the propagating beam direction almost coincides with z axis.

The isofrequency surfaces in Fig. 1 indicate that in some spatial directions there is no wave propagation, while in certain directions such media support propagating high-k waves^1^. Propagation is forbidden along *x* and *y* axes for I type media and along *z* axis for II type media. On the contrary, high-k wave propagation is supported in the directions close to the normal to the asymptote of the hyperboloid. This effect is used in tasks where strong localization of light is required.

Radio waves with frequencies up to the low edge of terahertz (THz) frequency range can be manipulated by means of artificial media on the basis of hyperbolic metamaterials^3^. These structures consist of subwavelength unit cells and are characterized by effective permittivity tensor. They are relatively difficult to manufacture and work only in a narrow frequency range specified by the unit cell parameters. Their application at infrared and visible wavelengths is an extremely difficult task due to physical limitations of production^5^. The problem can be solved using a special class of hyperbolic media on the basis of natural metal-like anisotropic materials with different plasma frequencies in orthogonal polarization directions^5^. Such materials have permittivity tensor components of different signs in a certain frequency range. They include Bi$_{2}$Se$_{3}$ and Bi$_{2}$Te$_{3}$ (tetradymites)^6^, MgB$_{2}$ and graphite^7^, hBN^8^, sapphire and Bi^9^, and others. It was shown theoretically that the simplest material with hyperbolic dispersion in the terahertz frequency range is bismuth^9^ which has semimetallic properties and which was well studied in recent works in a thin-film regime^10^. In the mentioned work, it was shown that bismuth film with a thickness more than 15 nm displays a continuous structure, and its dielectric function is comparable with that of bulk bismuth. Recently, the first thin-film-bismuth-based hyperbolic medium in the terahertz frequency range was demonstrated experimentally^11^. It was shown that properties of bismuth-dielectric bilayer structure strongly depend on Bi and dielectric thickness and permittivity. Dielectric plays an important role as a substrate for a thin film considered as such due to a high ratio of wavelength to film thickness. It was also shown that usage of Bi$_{1-x}$Sb$_{x}$ alloys with variable antimony concentration *x* instead of Bi films allows to tune the parameters of hyperbolic media^12^. In particular, at a certain antimony concentration, the semimetal-semiconductor transition occurs in Bi$_{1-x}$Sb$_{x}$ alloys^13^.

However, variation of Bi$_{1-x}$Sb$_{x}$ film thickness or antimony concentration in alloy as well as substrate material allows only to statically tune the effective hyperbolic dispersion of a film-on-substrate bilayer structure. The dynamical tuning vital for many tasks may be achieved through various mechanisms affecting film parameters such as influence of temperature, electric and magnetic fields, and optical pumping. Using such approaches, the elliptic-to-hyperbolic isofrequency surface topological transition may be attained^14^.

It should be emphasized that control of the topological transition in such anisotropic media has been demonstrated in the microwave and infrared frequency ranges^14–18^.

In this work, we perform experimental studies of an optically-induced switching between elliptic and hyperbolic permittivity dispersion of a semimetal-dielectric structure in the terahertz frequency range (II type hyperbolic medium). We show the possibility of realizing a simple structure with optical switching depending on the 980 nm optical pumping intensity. This structure works over a relatively wide frequency range due to the use of non-structured natural materials (bismuth and mica).

## Results

Using pulsed terahertz time-domain spectroscopy setup (see Methods), the influence of continuous-wave optical pumping (wavelength of 980 nm) of different power onto effective refractive index and permittivity of film-on-substrate structure was studied in 0.2-1.0 THz frequency range. 40 and 120 nm Bi films on the surface of 21 µm mica substrate (with an area of 80 mm$^{2}$) were evenly irradiated by optical pumping with a power from 0 to 600 mW with a step of 200 mW.

### Terahertz waveforms and complex amplitude spectra

**Figure 2:**
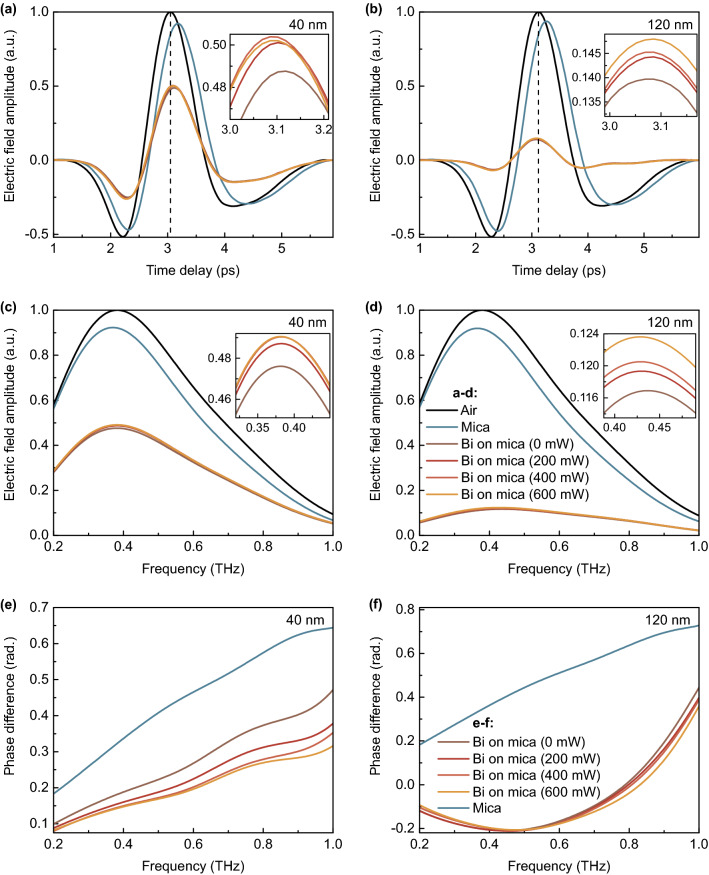
Results of experiment. Waveforms (**a,b**), amplitude (**c,d**) and phase difference (**e,f**, in relation to air signal) spectra of THz pulse transmitted through the mica substrate and the whole Bi-on-mica structures (with Bi thickness of 40 (**a,c,e**) and 120 (**b,d,f**) nm) which are influenced by optical pumping with different power.

Waveforms of THz pulse transmitted through the air, the substrate and the film-on-substrate structures (temporal dependencies of THz wave electric field amplitude) were recorded and filtered to exclude multiple reflections from further calculations (Fig. 2a,b). It can be seen that there is positive temporal shift of THz wave transmitted through the mica substrate which is associated with its positive permittivity. However, the whole film-on-substrate structures produce negative phase shift in the studied range of frequencies, as it will be shown below. It should be noted that the value of such temporal shift is higher in the case of thicker film, but the transmittance is significantly lower in this case too.

The recorded THz waveforms were then used to obtain corresponding amplitude (Fig. 2c,d) and phase (Fig. 2e,f) difference spectra (relative to phase spectrum in air) by Fourier transformation. With increasing optical pumping power, amplitude spectrum of THz wave transmitted through the whole Bi-on-mica structure grows insignificantly. At the same time, corresponding phase spectrum slightly decreases, showing a negative phase shift with respect to the substrate signal. Despite this, in the case of 40 nm films, phase shift of a THz wave transmitted through the whole structure is positive in relation to air signal. On the contrary, in the case of 120 nm films, this shift is strong enough and reaches negative values in the most of investigated frequency range.

### Effective parameters

On the basis of the obtained amplitude and phase spectra of a THz wave transmitted through the air and the whole structures, the complex effective refractive index dispersion was calculated using the next formula without considering the complex transmission coefficient^19^:

3 $$\begin{aligned} \hat{n}_{eff}(f)=\frac{c\left[ \phi _{whole}(f) - \phi _{air}(f) \right] }{2 \pi f d} + 1 - i \frac{c \ln {\left[ \left| \hat{E}_{whole}(f) \right| ^2 / \left| \hat{E}_{air}(f) \right| ^2 \right] }}{4 \pi f d}, \end{aligned}$$

where *c* is the speed of light in vacuum, *f* is the linear frequency of THz radiation, $\hat{E}_{whole}(f)$ and $\hat{E}_{air}(f)$ are the complex amplitudes ($\hat{E}=\left| E \right| e^{i \phi }$) of a THz wave passed through the whole structure and the air correspondingly, $\phi _{whole}(f)$ and $\phi _{air}(f)$ are corresponding phases, $d=d_{Bi}+d_{mica} \approx d_{mica}=21$ µm is the whole structure thickness, *i* is the imaginary unit.

However, since this formula does not take into account the complex transmission coefficient (which depends on both real and imaginary parts of $\hat{n}_{eff}$), the calculation is approximate and can give large errors in the case of significant absorption in the material. The iterative method^20,21^ was applied to obtain an exact solution for $\hat{n}_{eff}$ which would exactly agree with the results of the experiment (measured transfer function $\hat{H}_{measured}=\hat{E}_{whole}/\hat{E}_{air}$) or calculations based on the transfer matrix method^22^. The value of $\hat{n}_{eff}$ is corrected after each step of the iterative algorithm. The modeled transfer function is determined as

4 $$\begin{aligned} \hat{H}_{model}(f)=\frac{4 \hat{n}_{eff}(f) n_{air}}{\left[ \hat{n}_{eff}(f)+n_{air} \right] ^2} \cdot exp \left( -i \left[ \hat{n}_{eff}(f)-n_{air} \right] \frac{2 \pi f d}{c} \right) \cdot \hat{FP}(f), \end{aligned}$$

where $n_{air}=1$, the first term represents the complex transmission coefficient, and the last one takes into account the multiple reflections inside the sample (Fabry-Pérot effect):

5 $$\begin{aligned} \hat{FP}(f)=\left( 1 - \left[ \frac{\hat{n}_{eff}(f)-n_{air}}{\hat{n}_{eff}(f)+n_{air}} \right] ^2 exp\left[ -2 i \hat{n}_{eff}(f) \frac{2 \pi f d}{c} \right] \right) ^{-1}. \end{aligned}$$

Then, the errors were estimated for the amplitude and phase components as

6 $$\begin{aligned} ER_a(f)=\left| \hat{H}_{measured}(f) \right| - \left| \hat{H}_{model}(f) \right| ,\quad ER_p(f)=\angle \hat{H}_{measured}(f)-\angle \hat{H}_{model}(f). \end{aligned}$$

The complex effective refractive index was then updated:

7 $$\begin{aligned} Re(\hat{n}_{eff,new})=Re(\hat{n}_{eff,old})-s \cdot ER_p,\quad Im(\hat{n}_{eff,new})=Im(\hat{n}_{eff,old})-s \cdot ER_a, \end{aligned}$$

where *s* is the update step size, typically set to 0.01. The procedure is repeated until the correct value of the complex effective refractive index is obtained.

**Figure 3:**
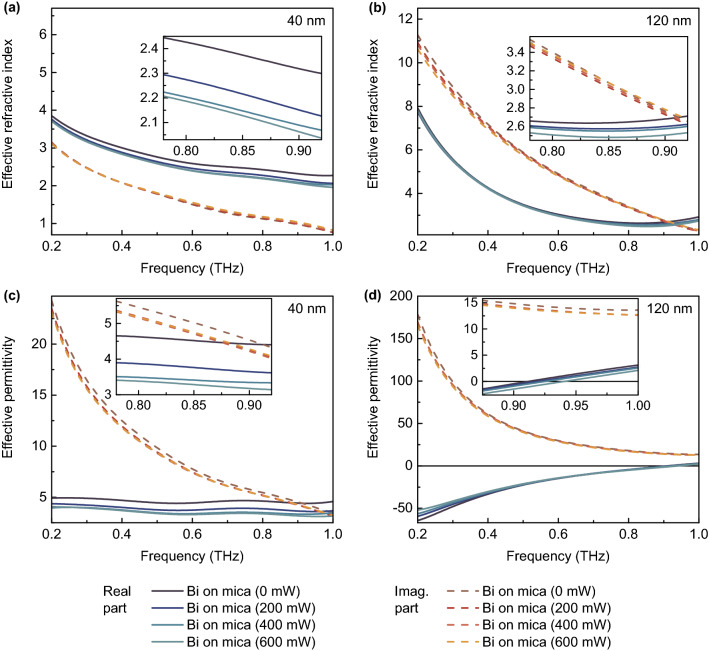
Results of experiment. Complex effective refractive index (**a,b**) and permittivity (**c,d**) dispersions of Bi-on-mica structures (with Bi thickness of 40 (**a,c**) and 120 (**b,d**) nm) which are influenced by optical pumping with different power.

Thus, in the case under study, there is a complex dependence of the transfer function on the complex refractive index and dependent parameters. The results of calculations are presented in Fig. 3a,b. The complex effective permittivity dispersion was calculated as $\hat{\varepsilon }_{eff}(f)=\hat{n}_{eff}^2(f)$ (see Fig. 3c,d).

The real part of the effective refractive index of Bi-on-mica whole structure is close in the cases of 40 nm and 120 nm bismuth films at higher frequencies and is much higher at lower frequencies in the case of 120 nm Bi. However, the imaginary part of the effective refractive index (which is responsible for losses) is much higher in the case of thicker film. The real part slightly decreases with rising optical pumping power in both cases, while the imaginary one changes insignificantly.

The effective permittivity of the whole Bi-on-mica structure is dramatically different in the case of 40 nm and 120 nm bismuth films. In the case of 40 nm Bi, the real part of the effective permittivity is positive, but decreases with rising optical pumping power. On the contrary, in the case of 120 nm Bi film, the effective permittivity is negative in almost the entire frequency range and passes through zero at a frequency of about 0.92 THz. Moreover, in the second case, the real part increases or decreases depending on the frequency of THz radiation under influence of optical pumping.

### Parameters of bismuth and mica

To understand the reasons for the presented behavior of the effective structure parameters, it is necessary to analyze the behavior of its components.

**Figure 4:**
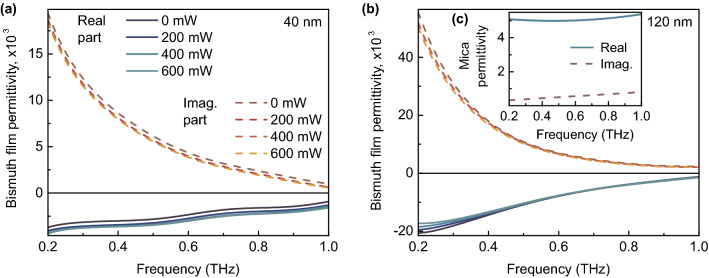
Results of experiment (material parameters). Complex permittivity dispersion of 40 (**a**) and 120 (**b**) nm bismuth films for various power of optical pumping. Inset: complex permittivity dispersion of mica substrate (**c**).

First of all, the initial value of the complex refractive index dispersion of the substrate was calculated as^19^

8 $$\begin{aligned} \hat{n}_{mica}(f)=\frac{c\left[ \phi _{mica}(f) - \phi _{air}(f) \right] }{2 \pi f d_{mica}} + 1 - i \frac{c \ln {\left[ \left| \hat{E}_{mica}(f) \right| ^2 / \left| \hat{E}_{air}(f) \right| ^2 \right] }}{4 \pi f d_{mica}}, \end{aligned}$$

where $\hat{E}_{mica}(f)$ is the complex amplitude of a THz wave passed through the mica substrate, $\phi _{mica}(f)$ is the corresponding phase. Next, the same iterative method (mentioned above) was applied to obtain an exact solution for the complex refractive index of the substrate. The optical pumping of the power used in the experiment does not affect the properties of the dielectric substrate. The calculated substrate complex permittivity dispersion ($\hat{\varepsilon } _{mica}=\hat{n} _{mica}^2$) is shown in Fig. 4c. It can be seen that losses in the substrate are small, and there is a relatively weak dispersion of the permittivity in the investigated frequency range.

Next, the in-plane complex sheet conductivity dispersion of the bismuth film, which is important for further calculations, was extracted using Tinkham’s formula (the film thickness is much smaller than a wavelength: $d_{Bi}/\lambda \le 4\cdot 10^{-4}$ for 120 nm films at 1.0 THz) as^23^

9 $$\begin{aligned} \hat{\sigma }_{Bi} (f)=[(\hat{n}_{mica}(f)+1)\hat{E}_{mica}(f)/\hat{E}_{whole}(f)-\hat{n}_{mica}(f)-1]/Z_{0}, \end{aligned}$$

where $Z_{0}$ is the free space impedance. The obtained conductivity dispersion was used to calculate the in-plane complex permittivity dispersion of the bismuth film as^24^

10 $$\begin{aligned} \hat{\varepsilon }_{Bi}=1+i \hat{\sigma }_{Bi} (f)/(2 \pi f d_{Bi} \varepsilon _{0}), \end{aligned}$$

where $\varepsilon _{0}$ is the absolute dielectric constant. The results of calculations are presented in Fig. 4a,b for 40 and 120 nm Bi films.

As it can be seen, Bi film permittivity strongly depends on its thickness due to the size effect, which is associated with carrier scattering on the material boundaries when the film thickness is decreased down to tens of nanometers^25^. In this case, low film thickness (about 250 nm or less at room temperature) reduces mean free path of charge carriers. In addition, crystallite size of the bismuth film is approximately proportional to its thickness, so the scattering of carriers at the borders of neighboring crystallites occurs in the studied case. The complex permittivity of a Bi film varies when changing the power of optical pumping, depending on its thickness and a frequency of THz radiation. In general, thin-film bismuth behaves like a metal when increasing optical pumping power. Theoretical study of thin-film bismuth parameters is complicated enough due to their strong dependence on film thickness, temperature, substrate material and morphology (affecting average size of crystallites and deformation of the film). The optical pumping changes the sample temperature affecting bismuth band structure^26^. It is important that sustained optical pumping with a high intensity (about 12.5 mW/mm$^2$ or more) may damage the sample since bismuth has a low melting point.

### Effective permittivity tensor

Thus, it is possible to calculate the diagonal components of a quasistatic permittivity tensor of the effective bismuth-on-mica structure using the effective medium theory^11,27^:

11 $$\begin{aligned} \hat{\varepsilon }_{xx}=\hat{\varepsilon }_{yy} \approx \frac{d_{Bi}\hat{\varepsilon } _{Bi}+d_{mica}\hat{\varepsilon } _{mica}}{d_{mica}}, \qquad \hat{\varepsilon }_{zz} \approx \hat{\varepsilon } _{mica}. \end{aligned}$$

The permittivity of the dielectric substrate is considered to be isotropic, while the calculation of $\hat{\varepsilon }_{xx}, \hat{\varepsilon }_{yy}$ requires only the in-plane bismuth permittivity $\hat{\varepsilon } _{Bi}$ (the orthogonal one does not affect any tensor components when $d_{Bi}/d_{mica} \ll 1$). In work of Elser et al.^28^, it was shown that in the case of metal-dielectric structure, the predictions of classical effective medium theory may not be accurate for a small number of periods. In the studied case of the only one period of a semimetal-dielectric structure, this error is associated with the non-local response and can be minimized using the nonlocal corrections $\hat{\delta }_{xx}$, $\hat{\delta }_{zz}$ derived in the mentioned work:

12 $$\begin{aligned}&\hat{\varepsilon }_{xx}^{nl}=\frac{\hat{\varepsilon }_{xx}}{1-\hat{\delta }_{xx}}, \qquad \hat{\delta }_{xx}=\frac{d_{Bi}^2 d_{mica}^2 \left( \hat{\varepsilon }_{Bi} - \hat{\varepsilon }_{mica} \right) ^2}{12 \left( d_{Bi} + d_{mica} \right) ^2 \hat{\varepsilon }_{xx}}\frac{\left( 2 \pi f \right) ^2}{c^2}, \end{aligned}$$

13 $$\begin{aligned}&\hat{\varepsilon }_{zz}^{nl}=\frac{\hat{\varepsilon }_{zz}}{1-\hat{\delta }_{zz}},\qquad \hat{\delta }_{zz}=\frac{d_{Bi}^2 d_{mica}^2 \left( \hat{\varepsilon }_{Bi} - \hat{\varepsilon }_{mica} \right) ^2 \hat{\varepsilon }_{zz}^2}{12 \left( d_{Bi} + d_{mica} \right) ^2 \hat{\varepsilon }_{Bi}^2 \hat{\varepsilon }_{mica}^2} \left( \hat{\varepsilon }_{xx}\frac{\left( 2 \pi f \right) ^2}{c^2} - \frac{k_z^2 \left( \hat{\varepsilon }_{Bi} + \hat{\varepsilon }_{mica} \right) ^2}{\hat{\varepsilon }_{xx}^2} \right) , \end{aligned}$$

where $k_z=2 \pi j / d$ for *j*-th mode (here $j=0$).

It is clear that the effective permittivity tensor components may be tuned by changing the ratio of the film and the substrate thicknesses. At a fixed frequency, an increase in the thickness of bismuth leads to an increase in the degree of anisotropy of the effective medium (at a fixed mica thickness), while an increase in the thickness of mica decreases this anisotropy (at a fixed bismuth thickness). It should be also considered that bismuth permittivity depends on its thickness because of the size effect on the nanometer scale.

**Figure 5:**
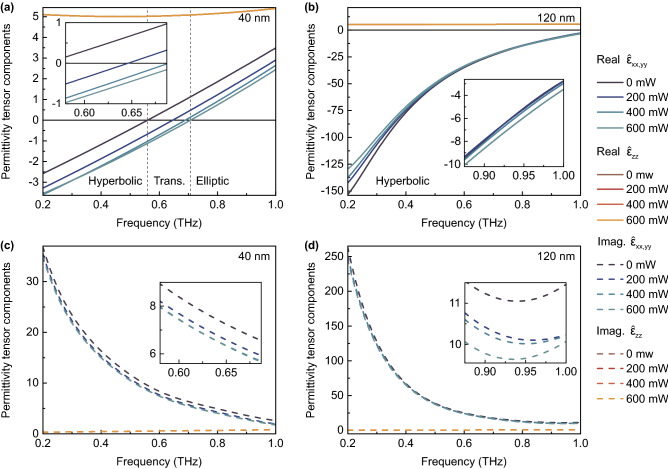
Results of experiment (permittivity tensor). Real (**a,b**) and imaginary (**c,d**) parts of permittivity tensor components of the whole Bi-on-mica structure in the cases of 40 (**a,c**) and 120 (**b,d**) nm bismuth films and for various power of optical pumping. The topological transition region is highlighted by vertical dashed lines in graph (**a**). The transition frequency point may be tuned in range from 0.560 THz to 0.708 THz.

The results of calculations are depicted in Figs. 5a,b and 5c,d for real and imaginary parts, correspondingly. Thus, for a fixed dielectric substrate thickness, the frequency position of the topological transition region depends on the bismuth film thickness. For the case of structure consisting of 40 nm Bi on 21 µm mica, the topological elliptic-to-hyperbolic transition occurs near 0.560–0.708 THz when changing power of the infrared continuous-wave optical pumping. In the case of 120 nm Bi on mica, the permittivity is only hyperbolic in the studied frequency range, but it can be also slightly tuned by optical pumping. Obviously, in the latter case, the topological transition occurs at frequencies slightly above 1 THz. Due to Drude-like permittivity dispersion of the bismuth film, the effective medium is described by a hyperbolic isofrequency surface below the topological transition frequency point and by an elliptical isofrequency surface above this point. It can be seen that the effective permittivity dispersion directly calculated on the basis of complex amplitude spectra (Fig. 3) lies in the range between positive (perpendicular, $\hat{\varepsilon }_{zz}$) and negative (in-plane, $\hat{\varepsilon }_{xx}, \hat{\varepsilon }_{yy}$) diagonal components of the effective permittivity tensor dispersion and therefore is not equal to any of them (it represents the average value of these dispersions). Optical pumping allows a relatively small modulation of the permittivity tensor at room temperature; therefore, to have topological transition in a certain frequency range, it is necessary to carefully select the thickness of the bismuth film or a dielectric substrate. The main tuning of the parameters occurs during optical pumping with a power in the range from 0 to 600 mW, and its further increase will not lead to significant changes, since saturation is observed.

**Figure 6:**
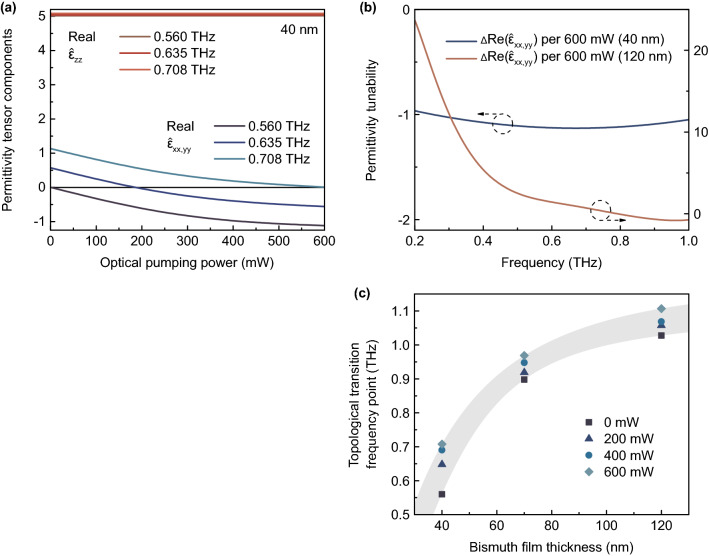
Results of experiment (tunable parameters). (**a**) Dependency of the real part of permittivity tensor components (40 nm Bi) on the optical pumping power for different frequencies of THz radiation. (**b**) The frequency-dependent tunability of $Re(\hat{\varepsilon }_{xx,yy})$ in the case of 40 and 120 nm Bi-based structures under influence of optical pumping with a maximum investigated power of 600 mW (7.5 mW/mm$^2$). (**c**) The dependency of topological transition frequency point (where $Re(\hat{\varepsilon }_{xx,yy})=0$) on bismuth film thickness (at a fixed mica thickness of 21 µm) for different optical pumping power values. The points on the graph correspond to the experimental values, and the highlighted area displays the assumed behavior according to the nonlinear dependence of bismuth dielectric permittivity on its thickness and THz radiation frequency. The points added for 70 nm Bi films are obtained on the basis of additional experiment with data presented in Supplementary information.

Figure 6a demonstrates the behavior of the permittivity tensor components (real part) in the case of 40 nm Bi. It is noteworthy that the elliptic-to-hyperbolic transition can be controlled with high precision by means of optical pumping, and $\hat{\varepsilon }_{xx}, \hat{\varepsilon }_{yy}$ can have a positive or negative value depending on the frequency of THz radiation. At the same time, $\hat{\varepsilon }_{zz}$ does not depend on the optical pumping power, but also depends on the radiation frequency. The frequency-dependent tunability of $Re(\hat{\varepsilon }_{xx,yy})$ is demonstrated in Fig. 6b for both 40 and 120 nm Bi-based structures under optical pumping with a maximum investigated power of 600 mW (7.5 mW/mm$^2$). The most interesting result is presented in Fig. 6c. This graph allows to select bismuth film thickness which is necessary to obtain a topological transition in a specific frequency range. For example, to have such a transition at a frequency of about 0.8 THz, it is needed to use 50 nm Bi film on 21 µm mica substrate.

The in-plane components of an effective permittivity tensor $\hat{\varepsilon }_{xx}, \hat{\varepsilon }_{yy}$ (which may be useful for various calculations) were fitted using classical Drude model:

14 $$\begin{aligned} \hat{\varepsilon }(\omega )=\epsilon _{\infty }-\frac{\omega _p^2}{\omega ^2+i \gamma \omega }, \end{aligned}$$

where $\omega =2 \pi f$ is the angular frequency of THz radiation, $\epsilon _{\infty }$ is the high-frequency permittivity, $\omega _p$ is the plasma frequency, and $\gamma$ is the damping frequency. The results of analysis are presented in Table 1. With rising optical pumping power, the high-frequency permittivity, plasma frequency and damping frequency tend to decrease, and the saturation of changes in these parameters were observed.

**Table 1:** Drude parameters. The in-plane effective permittivity tensor component in the case of 40 nm Bi vs. optical pumping power (OPP).

OPP (mW)	$\epsilon _{\infty }$	$\omega _p$ (THz)	$\gamma$ (THz)
0	6.89	2.515	0.791
200	6.16	2.394	0.742
400	5.79	2.355	0.727
600	5.61	2.359	0.736

### Isofrequency surfaces

**Figure 7:**
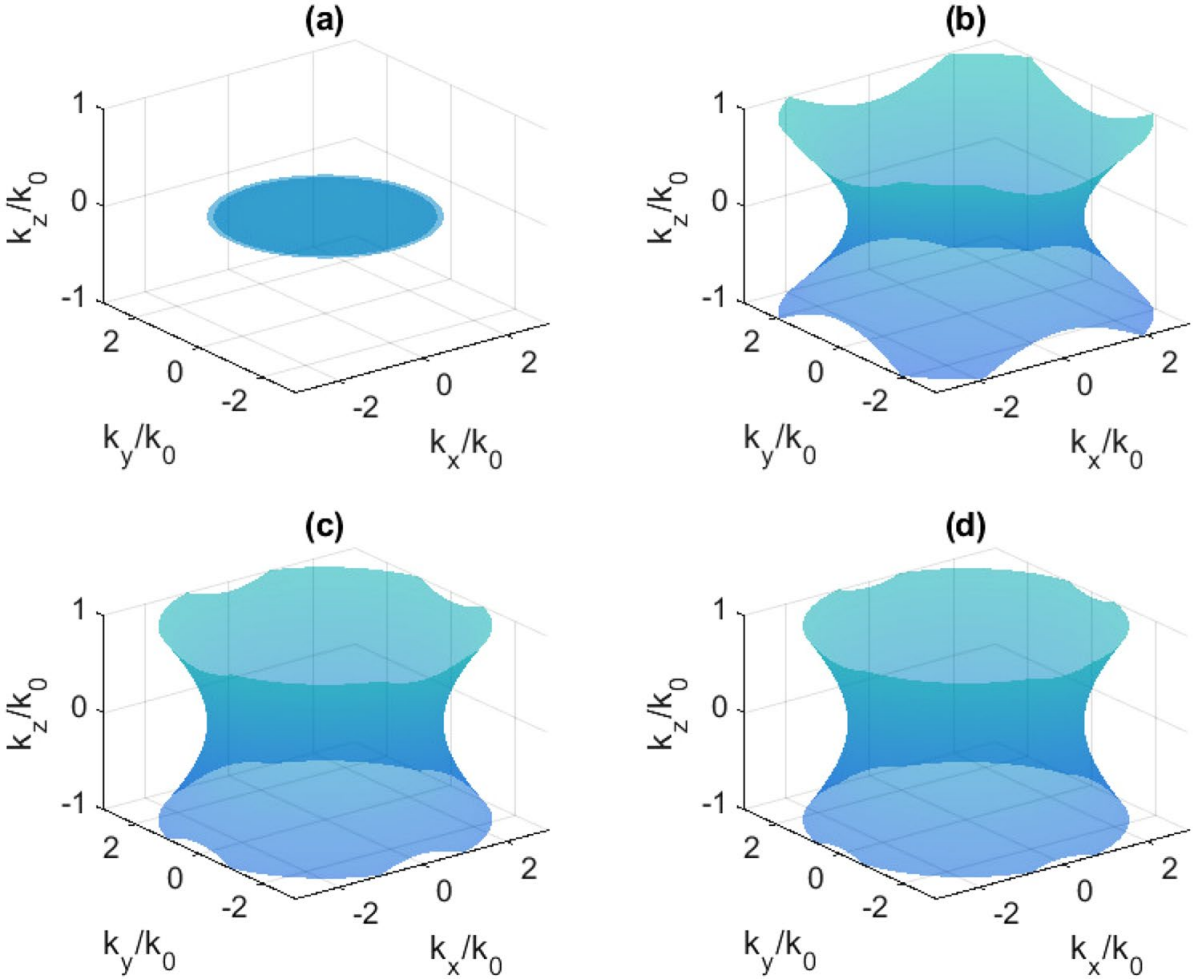
Isofrequency surfaces at 0.560 THz: (**a**) without pumping; (**b**) at 200 mW; (**c**) 400 mW; (**d**) 600 mW.

**Figure 8:**
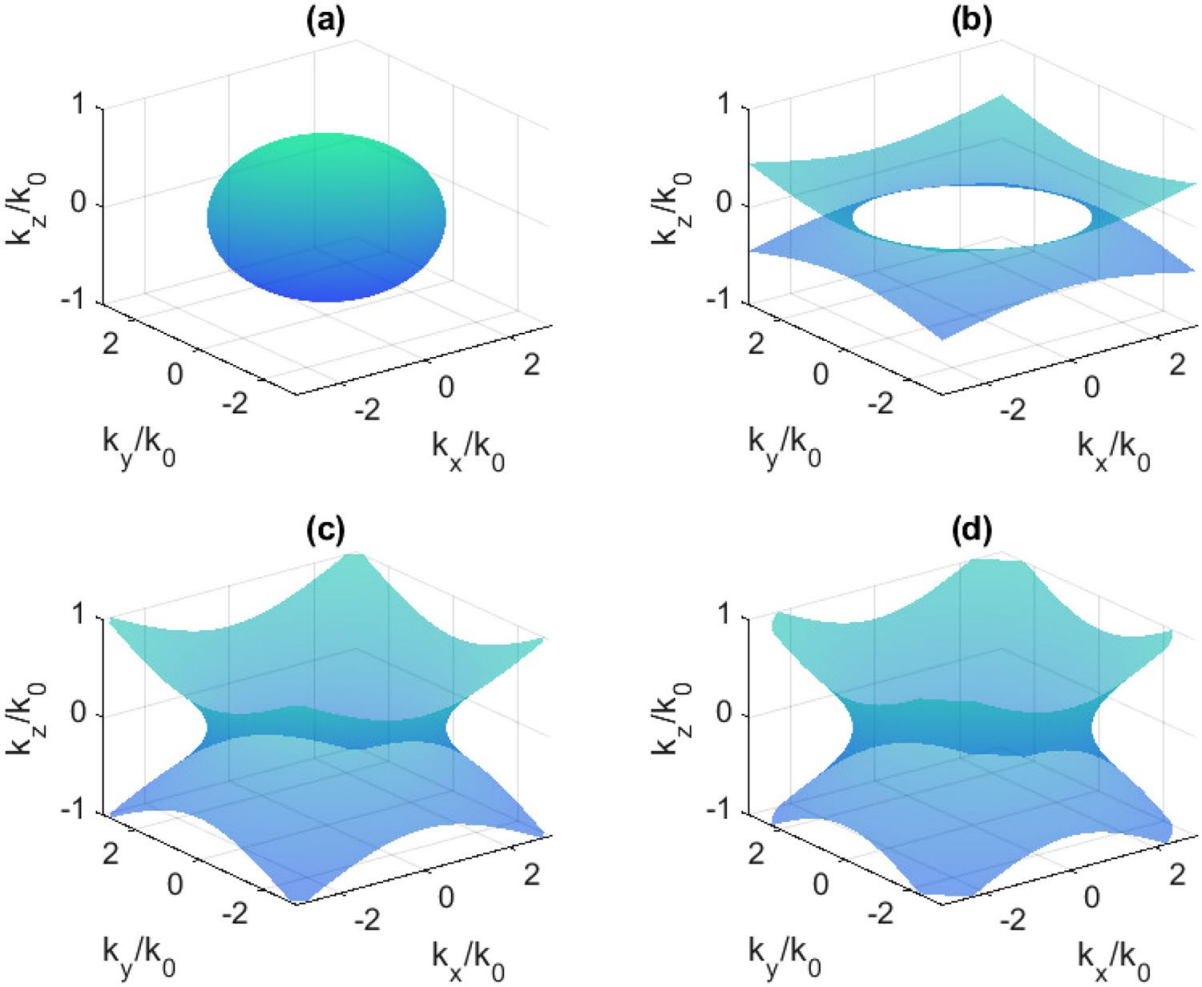
Isofrequency surfaces at 0.635 THz: (**a**) without pumping; (**b**) at 200 mW; (**c**) 400 mW; (**d**) 600 mW.

**Figure 9:**
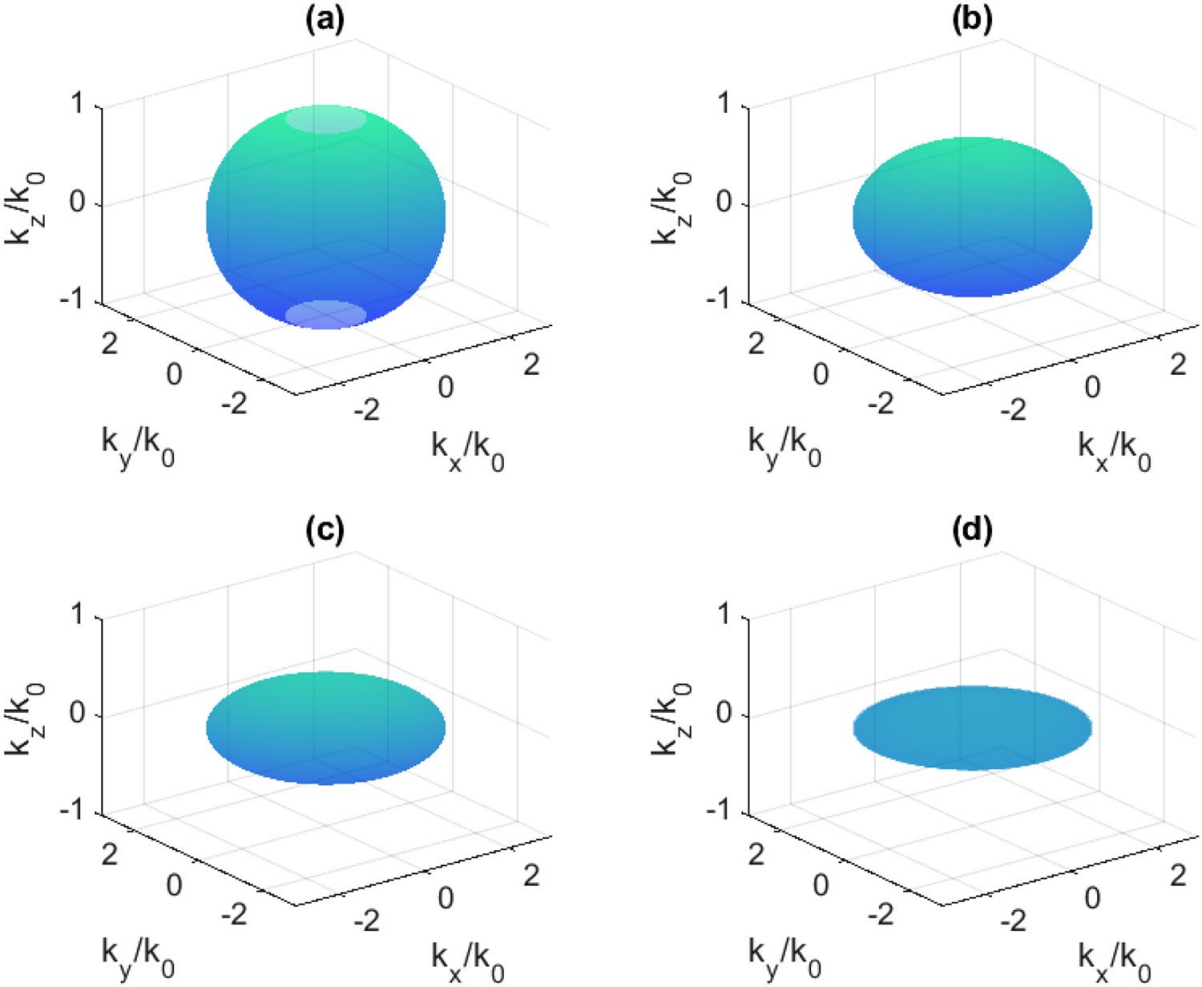
Isofrequency surfaces at 0.708 THz: (**a**) without pumping; (**b**) at 200 mW; (**c**) 400 mW; (**d**) 600 mW.

This transition results in a change of the isofrequency surface topology in reciprocal space, which is demonstrated in Figs. 7, 8, and 9 for the case of 40 nm Bi. It is obvious that the isofrequency surface is hyperbolic in the case of 120 nm Bi at any frequency in the investigated range.

The main factor producing the topological transition is the loss in the material controlled by infrared radiation^29^. In the presented case, these losses are associated with the imaginary part of $\hat{\varepsilon }_{xx}=\hat{\varepsilon }_{yy}$ (Fig. 5c,d). In work by Guo et al.^2^ it was mentioned that usually, topological transition is produced by changing the working frequency. However, at a fixed frequency, the intrinsic loss may be a parameter which determines such a transition. It was also said that the ability to actively tune these effects remains elusive and related experimental observations are highly desirable. In this work, we have experimentally demonstrated such a possibility using the simple bismuth-on-mica layered structure irradiated by 980 nm optical pumping of different power (different intensity). In a narrow frequency range, the optical pumping is the main switching factor. Switching does not take place in the entire frequency range of 0.2-1.0 THz, since a very wide range has been studied in which the bismuth permittivity is strongly dependent on the frequency.

## Applications

The optical pumping allows topological transition to be made controllable, paving the way for the development of all-optical devices with a tunable near-zero or hyperbolic permittivity dispersion in the THz frequency range. Despite the fact that a lot of work remains to optimize the operation of such a structure and to increase its efficiency, the principal possibility of this effect in the THz frequency range was demonstrated in this work for the first time.

The optically tunable structure consisting of a thin bismuth layer on the top of dielectric substrate is easy to produce and may be used in such applications as spontaneous emission enhancement^30^ (Purcell factor), tunable refraction^31^, sub-wavelength imaging^32^, sensing^33^, light confinement^34^, van Hove singularity^35^ and Compton^36^ effects.

## Methods

This section is devoted to fabrication and characterization of bismuth-mica structures and their studies by powerful method of terahertz time-domain spectroscopy. It allows to directly measure the amplitude and phase spectra of THz wave and thus to obtain an effective refractive index and effective permittivity spectra of such bilayer structures.

### Synthesis and characterization

To obtain thin bismuth films, the thermal spraying method in a vacuum^12^ was used. This method consists in converting the substance on the evaporator into a gaseous state and depositing it on a substrate located above the evaporator. The method of metered weighing was used to obtain a film of a certain thickness. During film growth, the substrate temperature was controlled. At the moment of bismuth deposition on the substrate, the temperature was 120 $^\circ$C, then annealing was carried out for 30 min at 250 $^\circ$C. During the entire experiment, the vacuum was $10^{-5}$  Torr. Then, the thickness of the obtained film was measured using Linnik’s interferometer MII-4, which has a measurement uncertainty of 5 nm. For further experiment, 40 and 120 nm bismuth films were obtained. Mica with a thickness of 21 µm was used as a substrate (one of the thinnest, but with a stable geometry and giving a possibility to synthesize high-quality bismuth film). The temperature, pressure and annealing affect the film quality and permittivity, but the synthesis of bismuth films was carried out under the optimal conditions mentioned above (according to work by Grabov et al.^37^ ). The film quality (average crystallite size) depends on its thickness.

**Figure 10:**
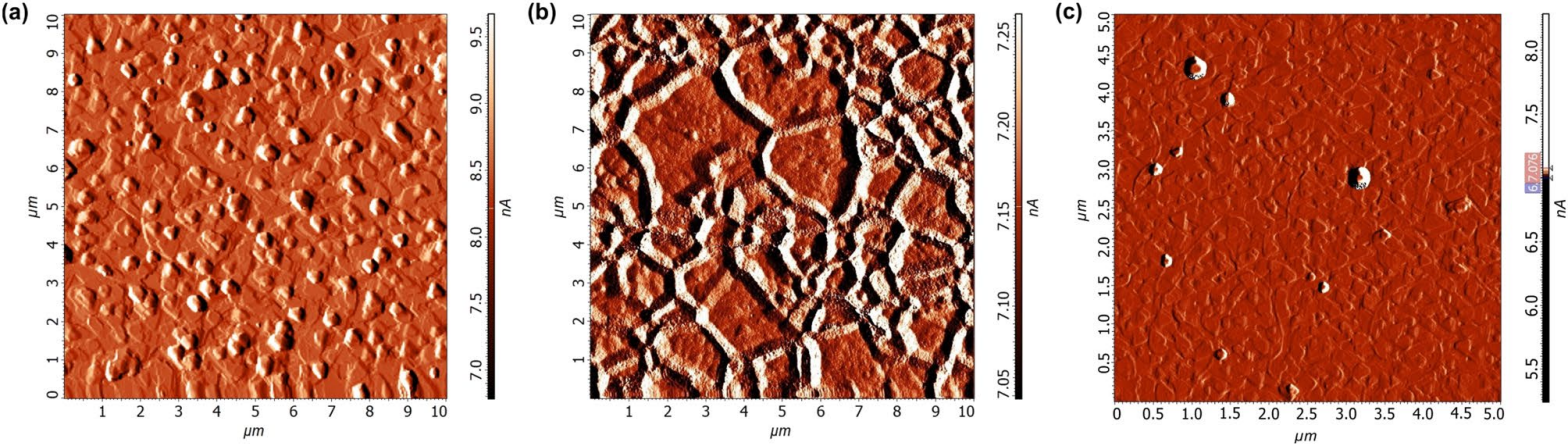
AFM-image of 120 nm Bi film surface before (**a**) and after (**b**) chemical etching. (**c**) AFM-image of 40 nm Bi film surface without chemical etching.

The film surface was studied in air using a Solver P47PRO scanning probe microscope (NT-MDT) by atomic force microscopy (AFM) in the semicontact mode. Cantilevers have a resonance frequency of about 150 kHz. Triangular growth patterns are visible on the film surface, which show that the $C_3$ crystallographic axis is directed perpendicular to the film plane (Fig. 10a,c). In one crystallite, the triangular growth figures have the same orientation; in the neighboring crystallites, the triangular growth figures have the opposite orientation. The orientation of the growth figures can be used to determine the boundaries and size of crystallites. Due to chemical etching (using a mixture of nitric and acetic acids taken in the volume ratio 1:200^38^), crystalline boundaries are more clearly visible and it is possible to characterize the size and shape of the crystallites more accurately (Fig. 10b).

In the studied case, the AFM is the best way to assess the thin film quality, because X-ray diffraction (XRD) gives too weak signal at a film thickness of 40 nm (practically only a substrate spectrum is visible). The scanning electron microscopy (SEM) may destroy the film and does not allow its further use. In addition, the electrons can not penetrate through the dielectric substrate in order to accurately determine the cross section of the bismuth-dielectric material. The detailed electrical measurements of various bismuth films^12,39,40^ allow assessing their quality, but not their morphology. In recent work^10^, it was stated that bismuth film with a thickness more than 15 nm displays a continuous structure, and its dielectric function is comparable with that of bulk bismuth. For a thickness lower than 15 nm, the film structure is discontinuous, and the dielectric function differs markedly from that of bulk bismuth. It is noteworthy that authors of the mentioned work use AFM and SEM to characterize the film structure and determine if it is continuous or discontinuous, but the AFM images have good enough quality to assess the film morphology. For discontinuous films, they propose FDTD simulation of nanospheroids separated by air gaps. However, since here we investigate 40 and 120 nm films, which are both continuous, the simulation method mentioned above is not required in the case under study.

### Terahertz time-domain spectroscopy

**Figure 11:**
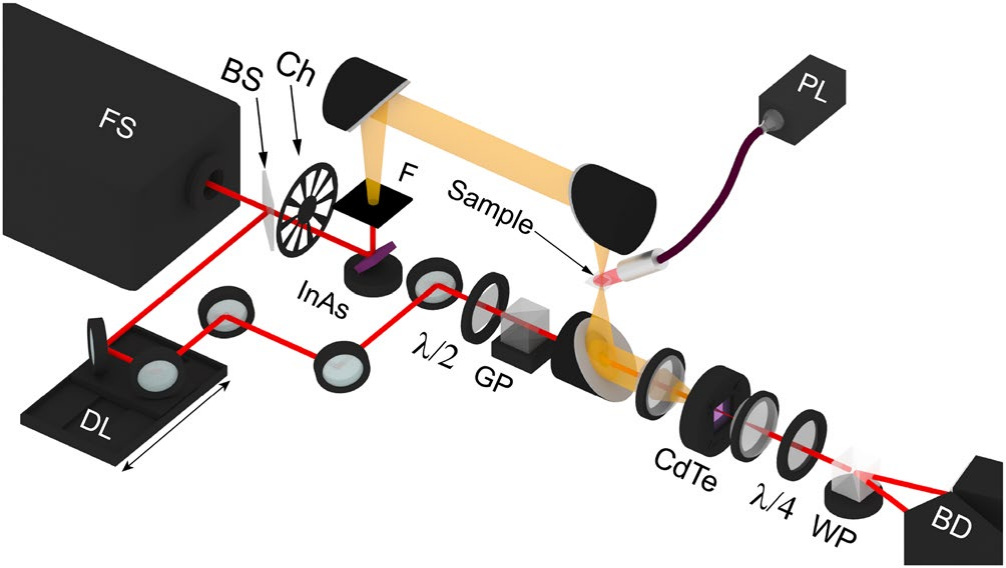
Schematic view of the THz time-domain spectroscopy setup working in transmission mode.

A schematic view of the terahertz time-domain spectroscopy setup^11^ is depicted in Fig. 11. A series of infrared pulses is generated by a femtosecond laser (1040 nm, 200 fs, 70 MHz, 15 nJ). The radiation is then divided by a beamsplitter into pump and probe beams. The first one passes through a time delay line and chopper and hits the indium arsenide (InAs) semiconductor placed in a magnetic field of 2 T which is used to generate THz radiation in the frequency range from 0.2 to 1.0 THz. This radiation passes through the sample which is placed in the focus plane and also irradiated by continuous-wave 980 nm optical pumping source and reaches the cadmium telluride (CdTe) semiconductor. The reflected radiation of the pump beam which propagates with the THz beam is cut off by an infrared filter. The probe beam passes through the polarizers, half-wave plate and Glan prism, and meets with the THz beam on the CdTe semiconductor surface. The ellipticity angle of polarization of the probe beam varies proportionally to the THz wave amplitude at a given time point, depending on the position of the time delay line. As a result, THz waveform is recorded by detecting the difference of intensities of orthogonally polarized components of the probe beam (which are divided by a Wollaston prism) using a balanced photodetector coupled with chopper (synchronous detection). The setup has a time resolution of 2 fs. The measurements were performed at a temperature of 290 K.

## Supplementary Information

Supplementary Information.
