# Supplementary material for: Experimental investigation of optically controlled topological transition in bismuth-mica structure
Source: Sci Rep. 2021 Jul 1;11:13653. doi: 10.1038/s41598-021-93132-9 (PMC8249560; doi:10.1038/s41598-021-93132-9)
Supplement: Supplementary file 1 — Supplementary Information. [file 41598_2021_93132_MOESM1_ESM.pdf]

Experimental investigation of optically controlled topological transition in bismuth-mica structure – Supplementary Information

Anton Zaitsev[1,*], Dmitry Zykov[1], Petr Demchenko[1], Mikhail Novoselov[1], Ravshanjon Nazarov[1], Maxim Masyukov[1], Elena Makarova[2], Anastasiia Tukmakova[2], Aleksei Asach[2], Anna Novotelnova[2], Natallya Kablukova[2], and Mikhail Khodzitsky[1]

[1]Terahertz Biomedicine Laboratory, ITMO University, St. Petersburg, 197101, Russia
[2]Faculty of Energy and Ecotechnology, ITMO University, St. Petersburg, 197101, Russia
[*]a.zaytsev@niuitmo.ru

S1. Results of THz time-domain spectroscopy of 70 nm Bi films on 21 μm mica substrate.

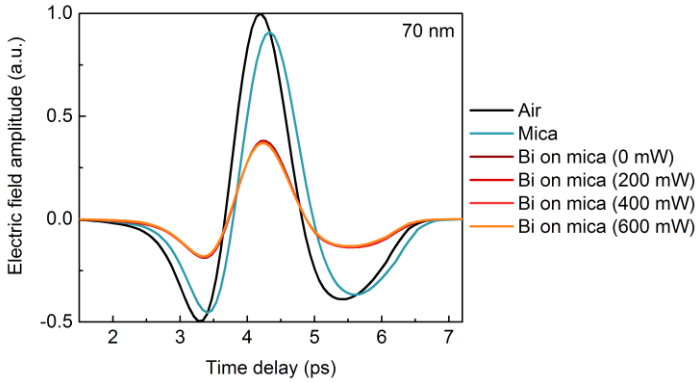

Figure S1. Waveforms of THz pulse transmitted through the mica substrate and the whole Bi-on-mica structures (with Bi thickness of 70 nm) which are influenced by optical pumping with different power.

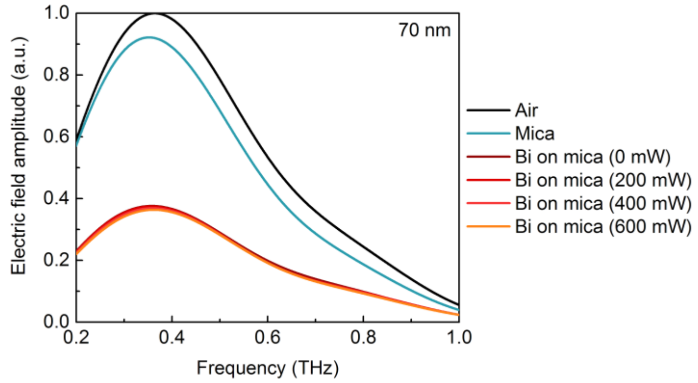

Figure S2. Amplitude spectra of THz pulse transmitted through the mica substrate and the whole Bi-on-mica structures (with Bi thickness of 70 nm) which are influenced by optical pumping with different power.

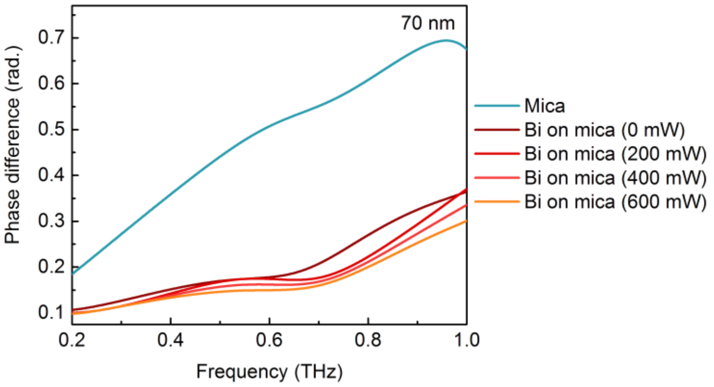

Figure S3. Phase difference spectra (in relation to air signal) of THz pulse transmitted through the mica substrate and the whole Bi-on-mica structures (with Bi thickness of 70 nm) which are influenced by optical pumping with different power.

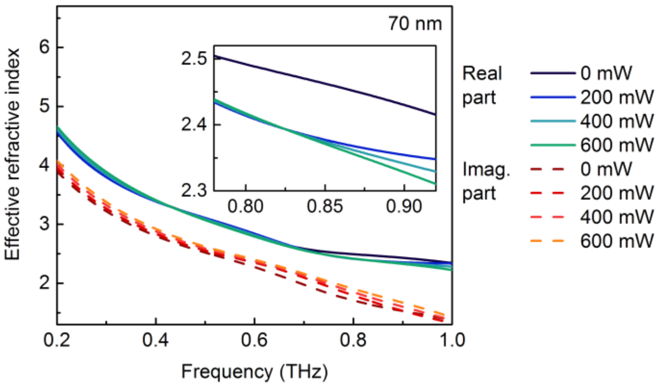

Figure S4. Complex effective refractive index dispersions of Bi-on-mica structures (with Bi thickness of 70 nm) which are influenced by optical pumping with different power.

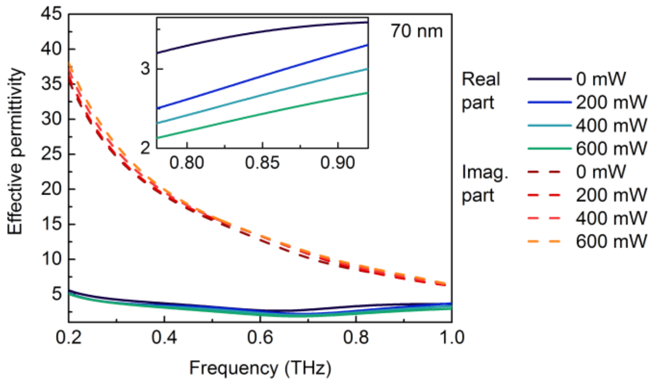

Figure S5. Complex effective permittivity dispersions of Bi-on-mica structures (with Bi thickness of 70 nm) which are influenced by optical pumping with different power.

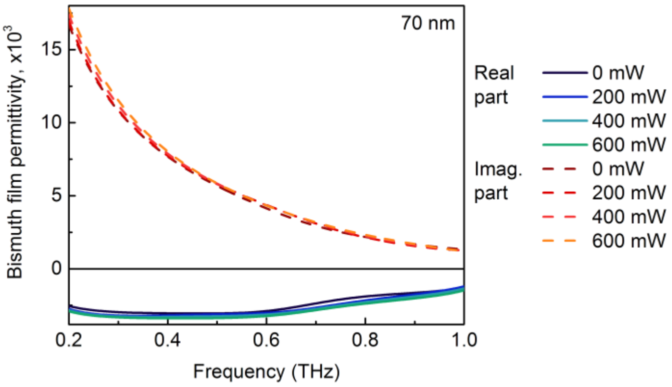

Figure S6. Complex permittivity dispersion of 70 nm bismuth films for various power of optical pumping.

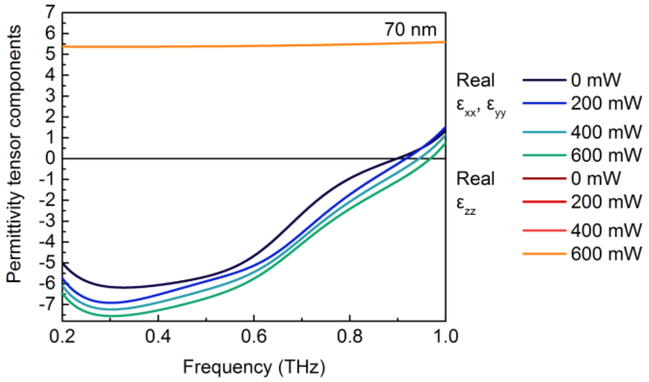

Figure S7. Real parts of permittivity tensor components of the whole Bi-on-mica structure in the case of 70 nm bismuth films and for various power of optical pumping. The topological transition occurs near 0.9 THz.

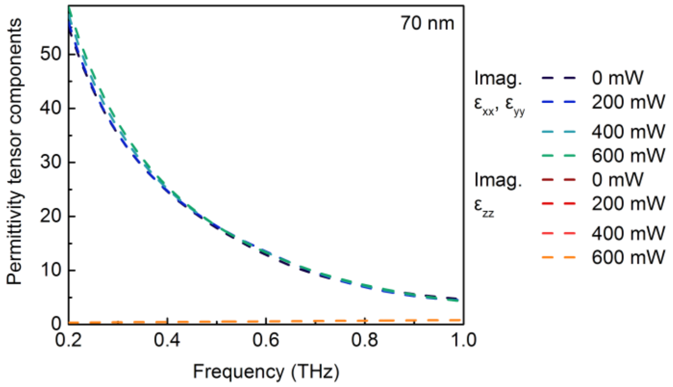

Figure S8. Imaginary parts of permittivity tensor components of the whole Bi-on-mica structure in the case of 70 nm bismuth films and for various power of optical pumping.
